# Effectiveness of conus lipoma surgery—a case series

**DOI:** 10.1007/s00381-025-06867-5

**Published:** 2025-06-09

**Authors:** Peter Spazzapan, Tomaž Velnar, Borut Prestor

**Affiliations:** 1 Clinical Department of Neurosurgery, Unit of Paediatric Neurosurgery, University Medical Centre Ljubljana, Ljubljana, Slovenia; 2 Clinical Department of Neurosurgery, University Medical Centre Ljubljana, Ljubljana, Slovenia; 3 Faculty of Medicine, University of Ljubljana, 1000 Ljubljana, Slovenia

**Keywords:** Tethered cord, Occult spinal dysraphism, Intraoperative neuromonitoring

## Abstract

**Introduction:**

The management of conus lipomas in children can be conservative, but prophylactic radical removal has been proven to be the only way to ameliorate the natural history of this disease. We started our surgical practice aiming toward total removal of spinal cord lipomas in 2017. This study aims to present our early results.

**Materials and methods:**

Patients younger than 12 years of age that had a conus lipoma surgically treated between April 2017 and April 2024 were included in the study. We analysed the preoperative presence of symptoms, defined the subtype of lipoma, the presence of syringomyelia and the degree of rotation of the placode. Among the operative data, we reviewed the preservation of the bulbocavernosus reflex (BCR). Postoperatively, we analysed the onset of new neurological or urological deficits and calculated the cord/sac ratio and the amount of residual fat.

**Results:**

A total of 19 patients have been included, with a median follow-up of 55.1 months. Dorsal lipomas were 21%, transitional 73.6% and chaotic 5.2% cases, while using the new classification of spinal lipomas (NCSL) type 1 were 63.1%, type 2 were 31.5%, and type 3 were 5.2%. Transitional and chaotic lipomas were significantly associated with a lower level of the conus (*p* = 0.0002), as type 2 and 3 of the NCSL (*p* = 0.0441). The patients were symptomatic in 31.5% and asymptomatic in 68.4% of cases. The preoperative presence of symptoms was associated with an age at surgery higher than 3 years (*p* = 0.0006). The median age at surgery was 65 months. The residual fat tissue was < 20 mm^3^ in 36.8%, 20–1000 mm^3^ in 47.3% and > 1000 m^3^ in 15.7%. A higher residual fat was associated with transitional and chaotic lipomas (*p* = 0.0082) and with types 2 and 3 (NCSL) (*p* = 0.0409). The median cord/sac ratio was 38.1%. After surgery, a permanent urinary deterioration occurred in 5.2% and a sensory deficit in 21%. The onset of permanent urological deficits was significantly associated with the loss of the BCR (*p* = 0.0012).

**Conclusions:**

Our results confirm the difficulty of achieving a safe and radical excision in transitional and chaotic (NCSL types 2 and 3) lipomas. These lipomas were significantly related to a higher postoperative amount of residual fat and to the occurrence of complications. For these lesions, the concept of radical resection should be shifted towards a concept of untethering by means of partial resection with maximal preservation of neurological function.

## Introduction

Conus lipomas can be classified using the conventional classification into dorsal, transitional, terminal and chaotic [1–4] and the new classification of spinal lipomas (NCSL) into types 1, 2 and 3 [5]. Conus lipomas typically tether the spinal cord, and while many infants may be asymptomatic at presentation, some of them present symptoms that affect the lower limbs and the sphincter function.

Historical controversy exists regarding the right therapeutic approach, which might be conservative or surgical. Conservative treatment has been historically advised because surgical results of prophylactic partial resection showed an even higher rate of late deterioration than the untreated disease [2, 6–10]. However, many authors [4, 11–14] believe that prophylactic surgery, particularly in infants and young children, would be the only way to ameliorate the natural history of the spinal cord tethering [4, 13, 14].

We started our surgical practice of radical removal of spinal cord lipomas in 2017. Our Unit of Paediatric Neurosurgery serves as a tertiary paediatric neurosurgical referral centre for whole Slovenia and covers a population of 400,000 children. The aim of this study was to compare our early results of resection of conus lipomas with those of other, larger studies [4, 15–18] and to review our outcomes.

## Methods

We performed a retrospective review of all patients surgically treated for a conus lipoma at our institution between April 2017 and April 2024. Since the clinical course after surgery is different between infants and late adolescents [16, 18] and in order to have a homogeneous cohort, we limited the study to patients younger than 12 years.

**Fig. 1:**
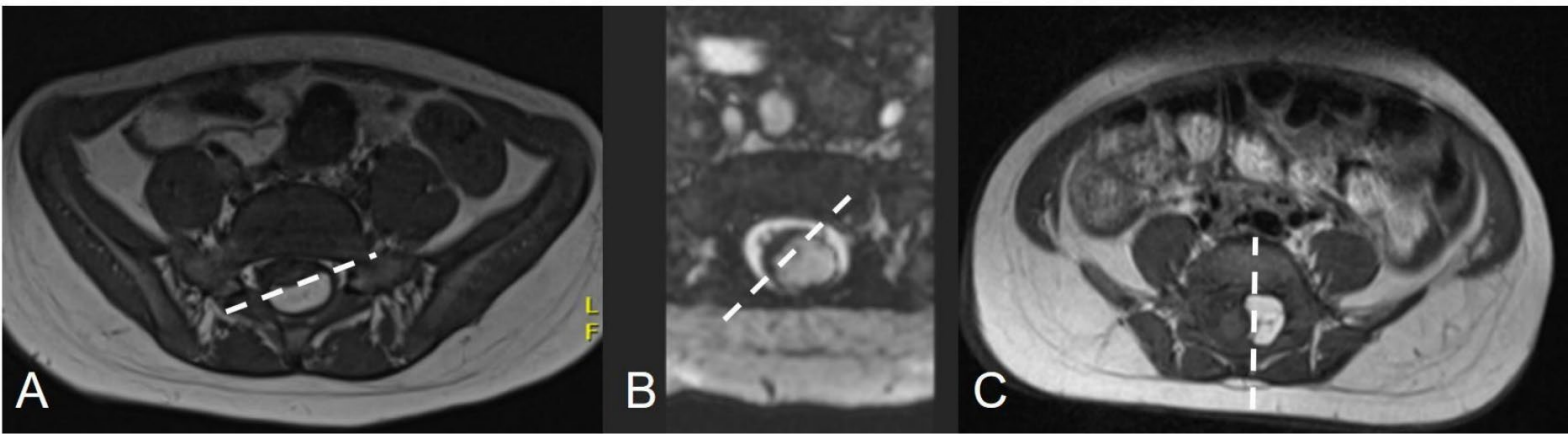
The surface between the neural and fat tissue can rotate to different degrees. The white dotted lines indicate three different levels of rotation of the placode. The rotation was classified in our series as 0° (**A**), 0 to 45° (**B**), and 45 to 90° (**C**)

We included the following preoperative clinical data: sensorimotor deficits and pain of the lower limbs, urological deficits and the presence of orthopaedic deformities. Among the preoperative MR data, we included the subtype of lipoma (conventional classification and NCSL), syringomyelia and the degree of rotation of the placode (0°, 0–45° and 45–90°) (Fig. 1). Types 4 lipomas of the NCSL were not included since they are located within the filum terminale.

**Fig. 2:**
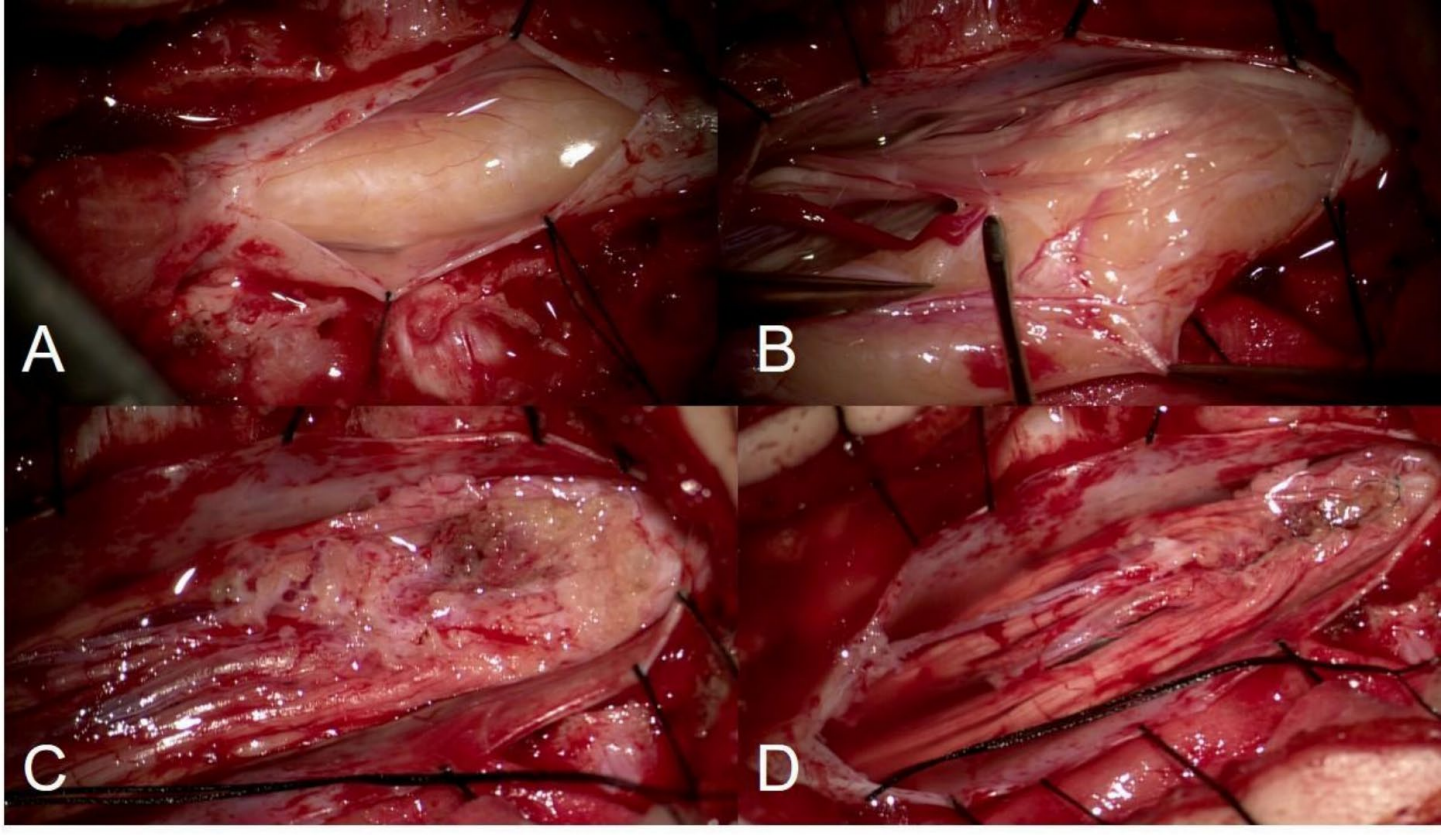
Intraoperative images showing the exposure of the lipoma through a cranial to caudal dural incision, toward the entrance of the lipomatous stalk through the dysraphic dura (**A**) and the dissection of the lipoma from the dura (**B**). The lipoma is then removed (**C**) leaving an irregularly shaped and ondulating white plane. In the end, the placode is neurulated (**D**) using a 7–0 monofilament suture

All patients have been operated by means of surgical principles previously described [4] and of an extensive use of intraoperative neuromonitoring (IONM). Surgery was indicated in all cases, irrespective of the morphology of the lipoma, but always after a deep and honest discussion with the family, explaining the benefits and risks of surgery, as well as the risks of a conservative approach. In all cases, the primary aim was a maximal safe resection (Fig. 2). IONM consisted of continuous monitoring of the spontaneous EMG activity of the perineal and lower limbs muscles and the bulbocavernosus reflex (BCR). Among the operative data, we reviewed the residual amplitude of the BCR at the end of surgery (> 50%, 25–50%, < 25%, loss of BCR).

**Fig. 3:**
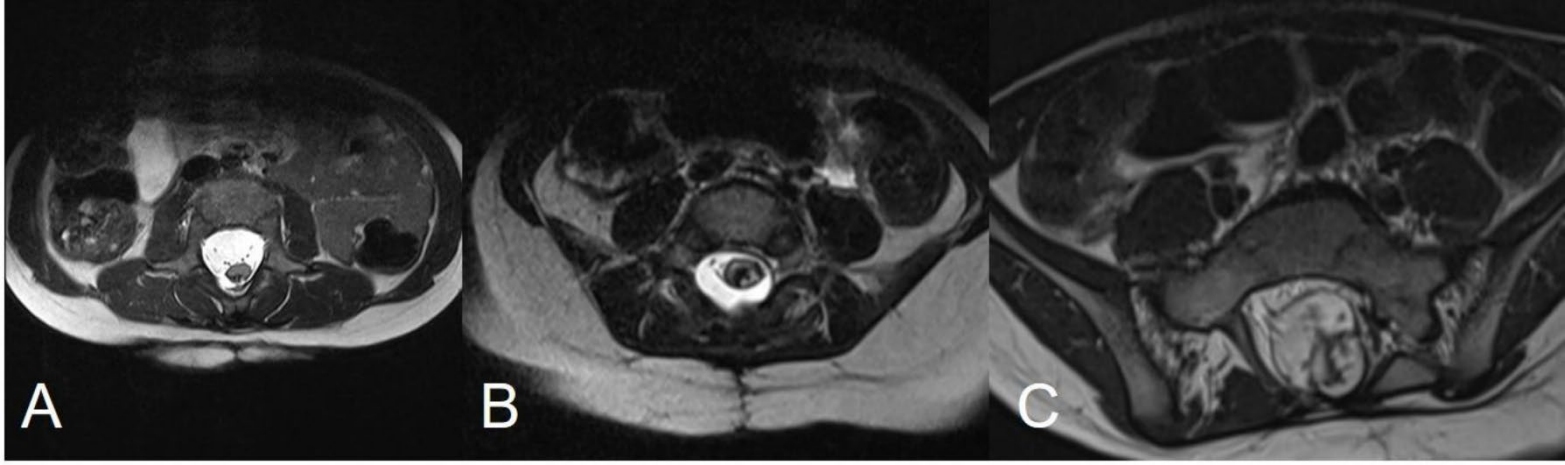
MR scans showing three different cord/sac ratios, obtained by dividing the diameter of the thickest portion of the neurulated placode in the axial plane by the diameter of the dural sac at the same level. A cord/sac ratio < 30% (**A**) indicates that the cord is located in a wide CSF bacine and well protected from potential retethering; 30–50% (**B**) and > 50% (**C**) indicate a progressively more limited space for the spinal cord within the reconstructed dural sac and thus a higher chance of retethering

**Fig. 4:**
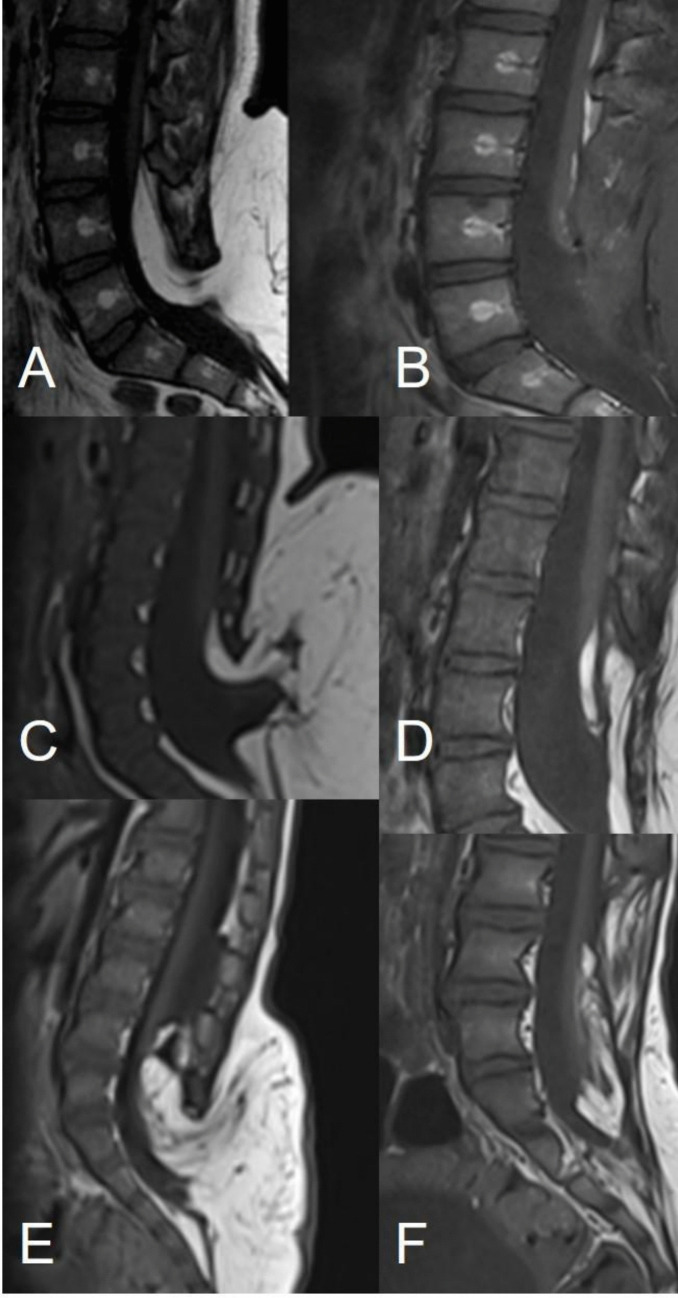
The image shows three different categories of residual fat. The transitional lipoma shown in image **A** has been totally removed (**B**) (residual fat < 20 mm^3^), the transitional lipoma in **C** has been subtotally removed (**D**) (residual 20–1000 mm^3^), while the fat residual after a subtotal removal of the transitional lipoma shown in image **E** exceeded the amount of 1000 mm.^3^ (**F**)

On the basis of the postoperative MR, we calculated the cord/sac ratio (< 30%, 30–50% and > 50%) by dividing the diameter of the neurulated placode in the axial plane by the diameter of the dural sac at the same level (Fig. 3). Furthermore, we divided the patients into three categories based on the amount of residual fat (< 20 mm^3^, 20–1000 mm^3^ and > 1000 mm^3^), calculated by multiplying the dimension of the residual fat tissue in the three planes on T1 sections (Fig. 4). Among postoperative clinical data, we included the onset of new neurological and urological deficits. The long-term results regarding retethering, development of scoliosis and other orthopaedic deformities were not included, due to a short follow-up.

Statistical analysis was done with the SPSS software version 18.0 (SPSS Inc., Chicago, IL, USA). A linear regression was used to define the relationship between two variables and estimate the value of a response. The significance level was *p* < 0.05.

## Results

### Preoperative data

A total of 19 patients have been included in the study (Table 1). The median follow-up was 55.1 months (min 17–max 91 months). Male patients were 6/19 (31.5%), female 13/19 (68.4%). A cutaneous stigmata were present in all cases. The median age at diagnosis was 39.5 months (min 0–max 11 years). The diagnosis was established in the first year of life in 13/19 (68.4%) cases. Conus lipomas were dorsal in 4/19 (21%), transitional in 14/19 (73.6%) and chaotic in 1/19 (5.2%) cases. Based on the NCSL, the lipomas were classified as type 1 in 12/19 (63.1%), type 2 in 6/19 (31.5%) and type 3 in 1/19 (5.2%). The conus was tethered at the level L3 in 4/19 (21%), L4 in 6/19 (31.5%), L5 in 4/19 (21%), S1 in 4/19 21(%) and S2 in 1/19 (5.2%) cases. At statistical analysis, a lower level of the conus was significantly associated with transitional and chaotic lipomas (*p* = 0.0002) and with NCSL type 2 and type 3 lipomas (*p* = 0.0441). This latter value was within the level of significance, but not strongly supported due to the small number of patients.

**Table 1:** Demographic, clinical, and radiologic data of the children included in the series

Male		6/19 (31.5%)
Female		13/19 (68.4%)
Cutaneous stigmata		19/19 (100%)
Median age at diagnosis		39.5 months
Diagnosis in the first year		13/19 (68.4%)
Subtype of conus lipomas (conventional classification)	Dorsal	4/19 (21%)
	Transitional	14/19 (73.6%)
	Chaotic	1/19 (5.2%)
Subtype of conus lipomas (NCSL)	Type 1	12/19 (63.1%)
	Type 2	6/19 (31.5%)
	Type 3	1/19 (5.2%)
Level of the conus	L3	4/19 (21%)
	L4	6/19 (31.5%)
	L5	4/19 (21%)
	S1	4/19 (21%)
	S2	1/19 (5.2%)
Syringohydromyelia		6/19 (31.5%)
Placode rotation	0°	13/19 (68.4%)
	0–45°	4/19 (21%)
	45–90°	2/19 (10.5%)

The placode was rotated for 0° in 13/19 (68.4%), 0–45° in 4/19 (21%) and 45–90° in 2/19 (10.5%) cases (Fig. 1). The rotation of the placode was not statistically associated with the subtype of lipoma, nor based on the conventional classification (*p* = 0.1780), nor on the NCSL (*p* = 0.375). A syringohydromyelia was present in 6/19 (31.5%) cases.

The patients were symptomatic in 6/19 (31.5%) and asymptomatic in 13/19 (68.4%) cases (Table 2). Symptoms and signs before surgery were paraesthesias in 5/19 (52.6%), pain in 3/19 (15.7%), motor deficits in 4/19 (21%), orthopaedic deformities in 4/19 (21%), scoliosis in 1/19 (5.2%) and urological deficits in 4/19 (21%) cases. Intermittent catheterisation was needed in 3/19 (15.7%) cases. The statistical analysis revealed a significant statistical correlation between the preoperative presence of symptoms and the age at surgery higher than 3 years (*p* = 0.0006). The preoperative presence of symptoms was not significantly associated with the amount of placode rotation (*p* = 0.289), nor with the presence of syringomyelia (*p* = 0.917), nor with the subtype of lipoma based on the conventional classification (*p* = 0.1408) and on the NCSL (*p* = 0.241).

**Table 2:** Presence of preoperative symptoms among the children included in the study

Asymptomatic patients		13/19 (68.4%)
Symptomatic patients		6/19 (31.5%)
	Paraesthesias	5/19 (52.6%)
	Pain	3/19 (15.7%)
	Motor deficits	4/19 (21%)
	Orthopaedic deformities	4/19 (21%)
	Scoliosis	1/19 (5.2%)
	Urological deficits	4/19 (21%)
	Intermittent catheterization	3/19 (15.7%)

### Intraoperative data

All patients have been surgically treated (Table 3). The median age at surgery was 65.1 months (min 3 months–max 11 years). The BCR was regularly elicitable at the beginning and at the end of surgery in 16/19 (84.2%) cases, while it has been lost (no residual amplitude) during surgery in 2/19 (10.5%) cases. The BCR was not recordable from the beginning of surgery in 1/19 (5.2%) case. The loss of the BCR was not significantly associated with the rotation of the placode (*p* = 0.378), nor with the subtype of lipoma based on the conventional classification (*p* = 0.468) and neither on the NCSL (*p* = 0.159).

**Table 3:** Operative and postoperative data

Median age at surgery		65.1 months
Bulbocavernosus reflex	Normal BCR at the end of surgery	16/19 (84.2%)
	BCR lost during surgery	2/19 (10.5%)
	BCR not recordable	1/19 (5.2%)
Residual fat	< 20 mm^3^	7/19 (36.8%)
	20–1000 mm^3^	9/19 (47.3%)
	> 1000 m^3^	3/19 (15.7%)
Subjectively total or near-total resection		15/19 (78.9%)
Subjectively partial resection		4/19 (21%)
Cord/sac ratio	Median cord/sac ratio	38.1%
	Ratio lower than 30%	4/19 (21%)
	Ratio between 30–50%	14/19 (73.6%)
	Ratio greater than 50%	1/19 (5.2%)
Surgical complications		4/19 (21%)
	Transitory urinary deterioration	1/19 (5.2%)
	Permanent postoperative deterioration	4/19 (21%)
	Permanent urological deterioration	1/19 (5.2%)
	Sensory deficits	4/19 (21%)

### Postoperative data

The median cord/sac ratio was 38.1%. The ratio was < 30% in 4/19 (21%), 30–50% in 14/19 (73.6%) and > 50% in 1/19 (5.2%) cases (Fig. 3). The cord/sac ratio was not significantly associated with the subtype of lipoma of the conventional classification (*p* = 0.494), nor of the NCSL (*p* = 0.583), nor with the amount of residual fat (*p* = 0.816).

The residual fat tissue was < 20 mm^3^ in 7/19 (36.8%), 20–1000 mm^3^ in 9/19 (47.3%) and > 1000 m^3^ in 3/19 (15.7%) (Fig. 4). Subjectively, based on the surgeon’s judgment, the resection was defined as total or near-total in 15/19 (78.9%) cases and partial in 4/19 (21%) cases. A higher residual of fat tissue was significantly associated with transitional and chaotic lipomas of the conventional classification (*p* = 0.0082) and with types 2 and 3 lipomas of the NCSL (*p* = 0.0409). Again, this latter value was statistically significant but not strongly supported due to the small number of patients in the cohort.

No progression or new onset of syringomyelia occurred during the follow-up period. A late deterioration due to retethering has not been detected in any case, and no second untethering procedure was performed. For this reason, the potential long-term protective effect of a low cord/sac ratio was not calculated.

### Complications

Surgical complications occurred in 4/19 (21%) cases: a wound infection in two cases, and a CSF fistula in two other cases. We have observed a transitory urinary deterioration in 2/19 (10.5%) cases. Overall, a permanent postoperative deterioration of the initial conditions occurred in 4/19 (21%) cases: a permanent urological deterioration in 1/19 (5.2%) case (mild urinary incontinence) and a new sensory deficit in 4/21 (21%) cases. The statistical analysis revealed a significant statistical correlation between the onset of permanent postoperative urological deficits and the complete loss of the BCR (*p* = 0.0012). There was no significant statistical correlation between the onset of permanent postoperative deficits and the subtype of lipoma, neither according to the conventional classification (*p* = 0.357) nor according to the NCSL (*p* = 0.406).

## Discussion

### Classification

As many as 33–60% of patients with conus lipomas are neurologically intact at presentation [15, 19, 20], but there is a tendency toward clinical deterioration over time [7, 19, 21]. This was also confirmed in our series, where children operated on after 3 years of age were more frequently symptomatic (*p* = 0.0006). The risk of deterioration, its timing and its pattern are in part related to the morphology of the lipoma and to the placode rotation. In addition, the NCSL defined the morphology of lipomas on the basis of the embryonic caudal neural tube formation and correlated these data with clinical findings and surgical complexity [5]. Our data confirm the similarities in clinical and radiological findings between transitional and chaotic lipomas of the conventional classification and type 2 and 3 lipomas of the NCSL.

### Treatment options

The treatment of conus lipomas has historically generated much controversy. Indeed, the work of Pang has changed the possibilities of surgical radicality [4, 13, 14], and his results were better than all previous surgical results, as well as the natural course of the pathology, as described so far. Nevertheless, the surgical results of many studies [15–18] still indicate that in some lipomas, a complete removal is not achievable. This was documented also by our series, where a subjective total resection was achieved in 78.9%. This discrepancy can be better understood by means of the NCSL, which introduced the concept of junctional neurulation to explain the pathogenesis of complex (type 2 and 3) forms of lipomas, in which the absence of the conus does not allow a radical resection [5, 17]. The question here arises: how much lipoma can be left behind to define the removal as radical? In the series from London, 43% of patients had > 20 mm^3^ residual fat [15], while in our series, this percentage was higher (63.1%), with 15.7% having an even higher residual fat of > 1000 mm^3^. Higher amounts of residual fat were observed in transitional and chaotic lipomas of the conventional classification and in types 2 and 3 lipomas of the NCSL**.** These data again confirm the complexity of achieving a radical resection, in particular in type 2 lipomas of the NCSL. Indeed, in these cases, the complex morphology carries high risks, and the intention of not causing any harm has often kept us in the layer slightly above the fusion line and the white plane. As a result, in several cases a thin layer of fat was left behind, which might have given the impression of a subtotal resection on postoperative MR. Furthermore, we observed that even a thin layer of residual fat can appear thicker on sagittal T1 cuts, due to the neurulation, which elevates this thin layer from a horizontal to a vertical plane.

The answer about how much fat can be left behind can be given by a low cord-sac ratio (Fig. 3). Achieving a low cord-sac ratio depends on the amount of residual fat, but also on a generous duraplasty [4]. We have achieved a median cord-sac ratio of 38%, while in the series from London [15], the median ratio was 47%. Pang reported a ratio < 30% in 72% of cases [4]. In our series, the protective effect of the cord/sac ratio from retethering has not been reported in our series, due to the relatively short follow-up period. Indeed, the diagnosis of retethering is predominantly clinical, and it cannot be established just on the basis of the MR, which often, even in our series, showed some contacts between the spinal cord and the surrounding dural sac. The reason might be related to the supine position of the patient during the MR, in which the conus moves toward the posterior wall of the dural sac. Performing MR in a prone position might help in distinguishing these cases from true attachments of the cord to the dural sac [22].

### Complications of treatment

Postoperative permanent neurological and urological deterioration occurs with a rate of 3–30% [23–25]. IONM undoubtedly helps to preserve neural structures, despite sensory rootlets, which are often “on the way” during the crotch dissection being less responsive to neurophysiological stimulation, compared to motor roots [26]. New postoperative sensorial deficits occurred in 21% of cases in our study, and all occurred in particularly complex lipomas. Furthermore, we found a statistical correlation between the onset of postoperative urological deficits and the complete intraoperative loss of the BCR (*p* = 0.0012). This confirms the criterion of “lost or remained” BCR amplitude, which can be used as a predictor of postoperative urinary function [27].

Although we did not confirm any statistical association between the appearance of postoperative deficits and the complex lipoma morphology (transitional and chaotic and type 2 and 3 according to the NCSL), we must notice that all permanent neurological deficits occurred in patients who had these subtypes of lipomas. This confirms the complexity of achieving a safe and radical removal of these lesions. Despite this, surgery remains important even in these cases, due to the benefit of untethering, compared with natural history. Indeed, the concept of radical resection should be approached with caution and moved to the concept of partial resection with maximal possible preservation of neurological function.

Our series included only children younger than 12 years, with the aim of having a homogeneous group. In this sense, the advantage of our series is to have a group of children of similar age and with a similar clinical and radiological baseline.

## Conclusions

In our study, the preoperative presence of symptoms was significantly associated with an age at surgery higher than 3 years. Complex lipomas (transitional and chaotic or types 2 and 3) were significantly associated with a lower level of the conus and with a higher postoperative fat residual. Furthermore, the intraoperative loss of the BCR was related to the onset of permanent urological deficits. Achieving a safe and radical excision in complex lipomas is technically difficult and carries a 21.7% risk of permanent neurological deterioration. In these cases, a conservative approach might be indicated, with an intervention only if neurological dysfunction appears.

## Data Availability

No datasets were generated or analysed during the current study.
